# BMP2 and VEGF165 transfection to bone marrow stromal stem cells regulate osteogenic potential in vitro

**DOI:** 10.1097/MD.0000000000009787

**Published:** 2018-02-02

**Authors:** Cong Zhang, Chunyang Meng, Dafan Guan, Fengyu Ma

**Affiliations:** a Department of Spine Surgery, Zhongda Hospital, Medical School of Southeast University, Nanjing, Jiangsu; b Department of Orthopedics, Affiliated Hospital of Jining Medical University, Guhuai Road, Jining, Shandong; c Department of Orthopedics, Ankang Central Hospital, Ankang, Shanxi, China.

**Keywords:** adenovirus vector, bone marrow mesenchymal stem cells, bone morphogenetic protein 2, bone regeneration, vascular endothelia growth factor 165

## Abstract

An exogenous supply of bone morphogenetic protein 2 (BMP2) and vascular endothelial growth factors 165 (VEGF165) will synergize to promote bone regeneration in vivo. The aim of this study was to confirm the role of VEGF165 on the osteogenesis potential of bone mesenchymal stem cells (BMSCs) transduced by adenovirus vector containing BMP2 gene in vitro.

Rabbit BMSCs were isolated and transfected with various adenovirus vectors: Ad-BMP2-VEGF165 (BMP2+VEGF165 group), Ad-BMP2 (BMP2 group), Ad-VEGF165 (VEGF165 group), and Ad-green fluorescent protein (GFP group). The multiplicity of infection was detected by GFP expression. Expression of BMP2 and VEGF165 was detected by Western blot and ELISA, and the osteogenic biological activity of BMP2 and VEGF165 by osteogenic assay. Meanwhile, the osteogenic biological activity of BMP2 and VEGF165 was evaluated by detection of Col I (collagen type I), OC (osteocalcin), and ALP (alkaline phosphatase) activity using OC staining, ALP activity assay, and real-time PCR assay.

Expression of target genes and proteins reached peak values at 5 days and then gradually declined. The OC staining, ALP activity, and real-time PCR assay of ColI, OC, and ALP were all increased in cells transfected with Ad-BMP2-VEGF165, Ad-BMP2, Ad-VEGF165, and Ad-GFP. However, the osteogenic biological activity in cells transfected with Ad-BMP2 was higher compared to cells transfected with other vectors after transfection at 14 and 21 days. We also found that BMP2 +VEGF165 group showed more osteogenic activity effect than the VEGF165 or control group. Furthermore, osteogenic assays in VEGF165 showed that a slightly lower osteogenic effect when compared to controls at 21 days.

VEGF165 might be a potent inhibitor of BMSCs differentiation into osteoblasts. The strategies to use BMP2 and VEGF165 in bone regeneration and the molecular mechanism of their interaction require further investigation.

## 1 Introduction

Large bone defects and nonunions arising from trauma, tumor, and other disease status are common problems faced by orthopedic specialists. They present a significant clinical challenge and lead to high costs for family and society.^[1]^ Autograft transplants serve as the “gold standard” for the treatment of osseous defects; however, bone regeneration is a complex process and the application of autograft inoculation results in some complications, such as nerves and blood vessel injury, risk of infection, and disease transmission.^[2]^

Gene therapy treatment to repair bone defects offers significant advances in regeneration medicine. Various growth factors have been verified for their capacity to promote bone healing, among which BMP2 and VEGF165 are among the most promising candidates.^[3]^ BMP2, one of the secreted proteins in the transforming growth factor-β superfamily, has high osteo-inductive activity^[4]^ and promotes bone repair in endochondral and membranous bones.^[5]^

Although BMP2 alone is sufficient to stimulate osseous formation, this highly coordinated process appears to involve various growth factors, including VEGF165, which promotes chemotaxis and proliferation of endothelial cells and play a pivotal role in bone regeneration.^[6]^ In addition, VEGF165 modulates chondrocyte apoptosis, cartilage remodeling, endochondral growth plate ossification, and osteoblast migration.^[7]^

BMP2 and VEGF165 can synergize to increase vascularization and promote bone regeneration. However, these growth factors have a short half-life, such that bolus delivery fails to promote proper bone and may result in ectopic bone formation and inflammation. The synergistic effect of BMP2 and VEGF165 on bone formation of tissue-engineered bones has been reported.^[8–10]^ However, the study of co-transfection of BMP2 and VEGF165 transgenes prevented ectopic bone formation in vivo compared to the BMP2 group was to be reported.^[11]^ Similarly, Li et al^[12]^ also found that expression of BMP2 and VEGF in pluripotent cells drastically decreases the potential of cell bone formation, but they did find an apparent reduction in BMP4 expression. Overexpression of VEGF165 inhibits BMP2 expression and MSC differentiation. The present study used adenovirus vectors co-expressing BMP2 and VEGF165 to determine their expression and osteoblast differentiation activity in vitro.

## 2 Materials and methods

### 2.1 Reagents

The adenovirus vector expressing BMP2 and expressing VEGF165 were kindly provided by Professor Wei Feng-Cai (Shandong University). DMEM/F12, fetal bovine serum, Percol (Gibco), trypsin, vitamin C, dexamethasone, DH5α, (Invitrogen), cDNA library (coming from hepar, human) and CsCl (Hanbio Technology Co., Shanghai), HEK-293 cells (Jika Gene, Shanghai), anti-CD14 APC (AbD serotec, UK), anti-CD29 PerCP and anti-CD44 PerCP (Biolegend), CD34 FITC (Miltenyi Biotec, Germany, isotype Ig as negative control antibody (Santa Cruz), Mouse antirabbit VEGF165, Mouse antirabbit BMP2, HRP-labeled secondary antibodies (Santa Cruz).

### 2.2 Preparation for BMSCs and Ad-BMP2-VEGF165

The third passage BMSCs and Ad-BMP2-VEGF165were stored in our prior study.^[13]^ The recombinant adenoviral vectors co-expressing BMP2 and VEGF165, which were E1 and E3 deficient recombinant adenovirus propagated in 293 cells through PAd-CMV-BMP2-VEGF165 co-transfection. The vectors were purified by cesium chloride gradient ultracentrifugation and the titers calculated with the value of OD260. All experiments were reviewed and approved by the Institutional Animal Care and Use Committee in Southeast University School of Medicine, and were conducted according to the committees’ guidelines.

### 2.3 Adenovirus vector construction, virus production and transduction

There were 4 groups in this study: Ad-GFP group (control), Ad-BMP2 group, Ad-VEGF165 group, and Ad-BMP2-VEGF165 group. BMSCs were transfected with Ad-GFP, Ad-BMP2, Ad-VEGF165, and Ad-BMP2-VEGF165 at various multiplicity of infection (MOI) of 60, 80, 100, and 120, respectively. The efficiency of adenovirus was quantitatively determined by GFP expression, which was observed by fluorescence microscopy after transduction at 48 hours. The best MOI (Ad-GFP is 100, Ad-BMP2, and Ad-VEGF165, are 100, Ad-BMP2-VEGF165 is 80) were obtained with the highest transfection efficiency up to 90%, while minimal apoptosis and morphological change were observed.

### 2.4 Western blotting analysis of BMP2 and VEGF165

BMSCs were transfected and incubated for 5 days. BMSCs were lysed with radio immune precipitation assay (RIPA) buffer with phenylmethanesulfonyl fluoride (PMSF) on ice for 10 minutes. Protein concentration was assessed, and 50 μg of protein was subjected to sodium dodecyl sulfate polyacrylamide gel electrophoresis. The separated proteins were transferred to a PVDF transfer membrane. Subsequently, the membranes were blocked for 1 hours at room temperature with blocking buffer. Then, antibodies against BMP2 or VEGF165 monoclonal immunoglobulin G (dilution 1:200; Santa Cruz) were incubated on the membrane overnight at 4°C and detected using secondary horseradish peroxidase conjugated antibodies (dilution, 1:2000; Santa Cruz) at room temperature for 1 hours. Finally, the PVDF transfer membrane was washed with Tris-buffered saline (1 × TBS) and developed using an enhanced chemiluminescence detection system.

### 2.5 Enzyme linked immunosorbent analysis of BMP2 and VEGF165

The levels of BMP2 and VEGF165 secreted from transfected BMSCs were determined by BMP2 ELISA Kit and VEGF ELISA Kit, respectively. The supernatant of the groups was harvested at the 1, 3, 7, 9, 11, 21, 28 days after virus transduction. Each group has 6 replicated wells at the different time. The assay was performed according to the manufacture's introductions. The medium was changed every 24 hours during the detection period.

### 2.6 Alkaline phosphatase activity detection

The BMSCs of each group were plated into 96 well plates at a density of 4 × 10^3^ cells per well and seeded in osteogenic differentiation medium, which containing 50 μg/mL ascorbic acid, 10^–7^ M dexamethason, 10 mM β-glycerolphosphate. The cells were analyzed for osteogenic differentiation at 7, 14, and 21 days with 6 replicated wells according to the ALP kit introduction. The reaction was stopped by adding stopping buffer (0.2 mol/L NaOH), and the absorbance was detected by a spectrophotometer at 450 nm.

### 2.7 Immunohistochemical detection for OC

BMSCs were cultured in osteogenesis conditioned medium. The effect on osteogenic induction was determined at 7, 14, and 21 days by immunohistochemical assay through OC staining. The cells were plated on a cover slip and culture medium replaced every 3 days. Cells were fixed with acetone with aminopropyltriethoxysilane (APES) for 20 minutes at room temperature and then incubated for 20 minutes at room temperature with goat serum to block the OC antigen. Monoclonal antibody against OC was added on the glass and incubated overnight at 4°C. The HRP-labeled secondary antibody was added to the specimens and incubated for 30 minutes at 37°C in a black humidity chamber. Nuclei counterstained with hematoxylin and the glass mounted with neutral resin. The integral density of positive cells was analyzed by the image Pro Plus Software.

### 2.8 Real-time polymerase chain reaction analysis

The 4 groups of cells were seeded in condition medium and mRNAlevels analyzed at 7, 14, and 21days. The 3 genes related to osteogenesis, including ALP, Col I, OC, were analyzed and the primer pairs were designed by using Primer3 software (Table 1). The cells were collected and total RNA extracted with RNeasy mini column. The extracted RNA was treated with DNase I to remove the DNA contamination, and the first strand cDNA was synthesized by random primer and oligo T and real-time PCR was performed using SYBR Green I. Thermal cycling parameters were as follows: 95°C for 30 seconds for 1 cycle, and then 40 cycles of 95°C for 5 seconds, 60°C for 30 seconds. A 2-ΔΔ^Ct^ method was used to evaluate the relative association of mRNA for each gene.

**Table 1:**
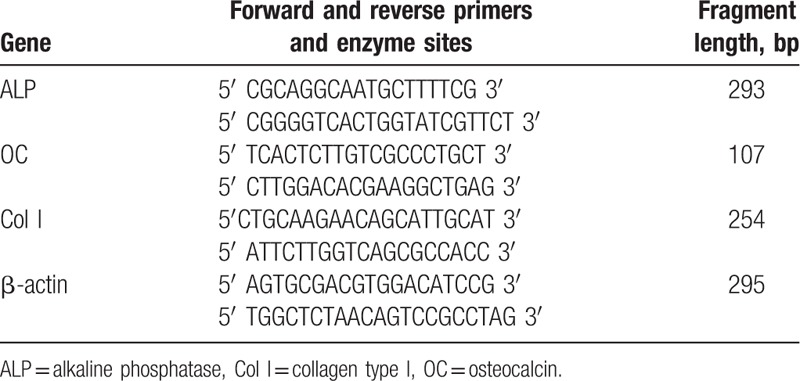
Description of the designed primers.

### 2.9 Statistical analysis

The data (Western blotting, ELISA, q-PCR, ALP activity detection, and immunohistochemical detection) were presented as mean ± SD and statistical significance was done by analysis of variance (ANOVA) using SPSS17.0 software. Statistical significance was set when a *P*-value less than .05.

## 3 Results

### 3.1 BMP2 and VEGF165 proteins expression in vitro

**Figure 1:**
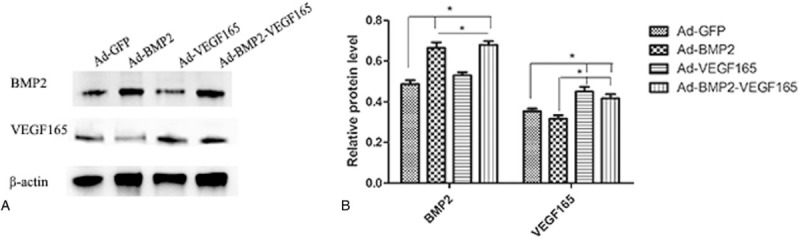
Protein expression of BMP2 and VEGF165 in each group on day 5. (A) BMSCs transfected with Ad-BMP2 and Ad-BMP2+ VEGF165 secreted large quantities of BMP 2. Meanwhile, the GFP group and VEGF165 group showed lower quantities BMP2 than above groups. (B) Histogram shows average of relative levels of proteins normalized to β-actin. BMP2 = bone morphogenetic protein 2, BMSCs=bone mesenchymal stem cells, GFP = green fluorescent protein, VEGF165 = vascular endothelial growth factors 165.

**Figure 2:**
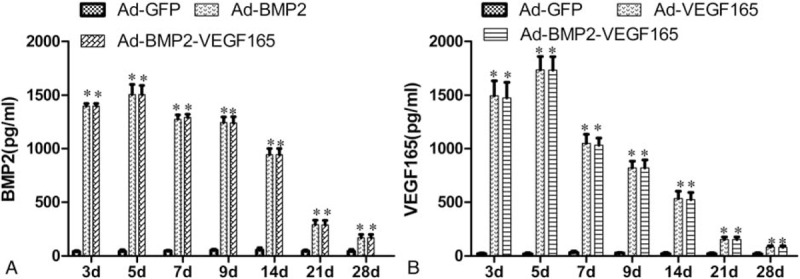
BMP2 and VEGF165 concentrations in harvested media samples from BMSCs of each group at 3, 5, 7, 9, 14, 21, and 28 days. (A) ELISA for BMP2. The BMP2 group and BMP2+ EGF165 group showed markedly BMP2 concentrations. ∗*P* < .05 compared with GFP group. (B) ELISA for VEGF165. The VEGF165 group and BMP2 + VEGF165 group showed markedly VEGF165 concentrations. ∗*P* < .05 compared with GFP group. Each assay was performed in triplicate. BMP2 = bone morphogenetic protein 2, BMSCs = bone mesenchymal stem cells, GFP = green fluorescent protein, VEGF165 = vascular endothelial growth factors 165.

Detection of BMP2 and VEGF165 proteins expression in vitro was performed by Western blotting and ELISA analysis as demonstrated in Figure 1. BMSCs transfected with Ad-BMP2 and Ad-BMP2-VEGF165 secreted large quantities of BMP2; however, the GFP group and VEGF165 group showed lower quantities BMP2 than above. On the other hand, the VEGF165 group and BMP2-VEGF165 group presented higher quantities of VEGF165 than the BMP2 group and control group. Proteins of interest in the transfected group peaked at 5 days and then reduced from 7 to 28 days, the data detected by ELISA for BMP2 and VEGF165 shown in Figure 2.

### 3.2 Immunohistochemical detection of OC

**Figure 3:**
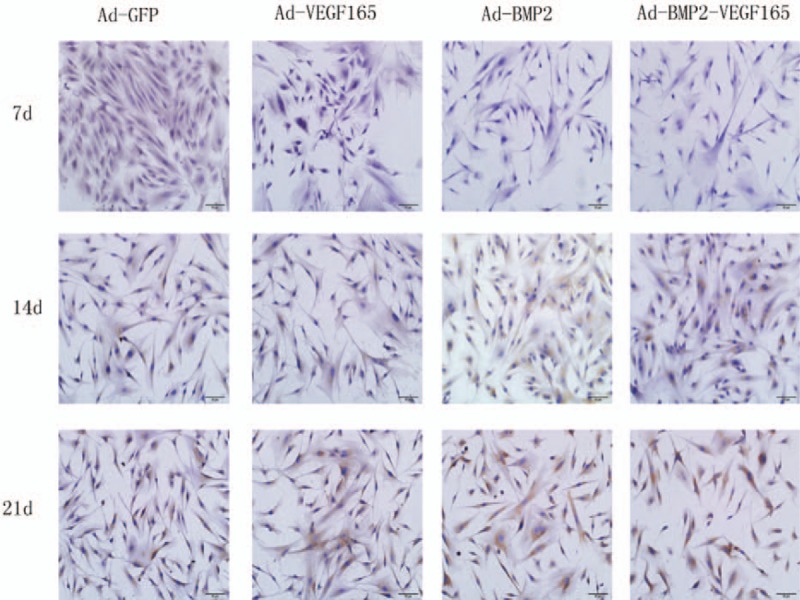
Osteocalcin staining in each groups at 7, 14, and 21 days and increased time dependently subsequent to transfection of BMSCs (scale bar = 50 m). Each assay was performed in triplicate. BMSCs = bone mesenchymal stem cells.

**Figure 4:**
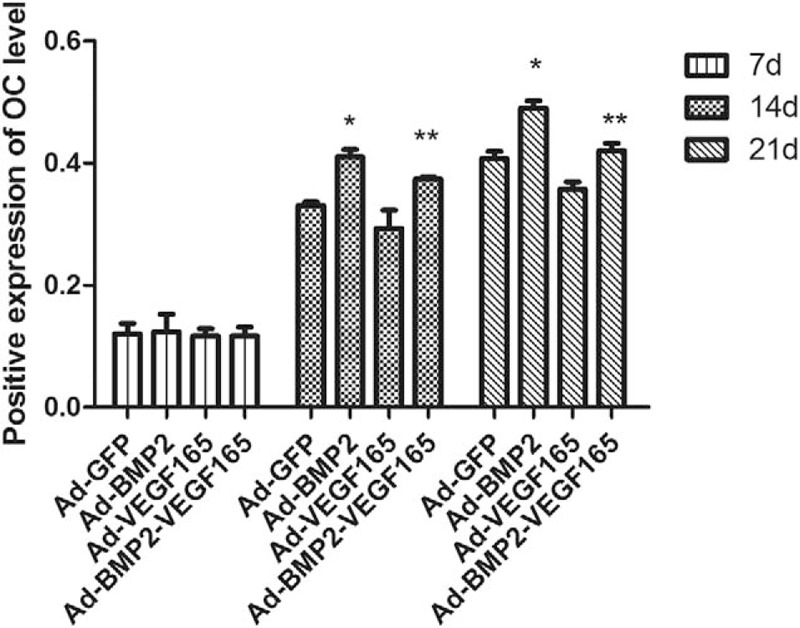
Positive expression of OC staining in each groups at 7, 14, and 21 days. The positive expression of OC in the BMP2 group was significantly higher among the other groups. ∗*P*<.05 compared with BMP2 +VEGF165 group, VEGF165 group, and GFP group. Meanwhile, the OC staining in the control group was significantly larger than that in the VEGF165 group and smaller than that in the BMP2+VEGF group at 14 and 21 days.∗∗*P*<.05 compared with VEGF165 group and GFP group. BMP2 = bone morphogenetic protein 2, GFP = green fluorescent protein, OC = osteocalcin, VEGF165 = vascular endothelial growth factors 165.

As shown in Figure 3, the positive expression of OC in the BMP2 group was significantly highest among the other groups at the 14 and 21 days (*P*<.05). Meanwhile, the OC staining in the control group was significantly larger than that in the VEGF165 group and smaller than that in the BMP2+VEGF group at 14 and 21 days. There was no significant difference between groups at the 7th day (*P* > .05) (Fig. 4)

### 3.3 ALP activity

**Figure 5:**
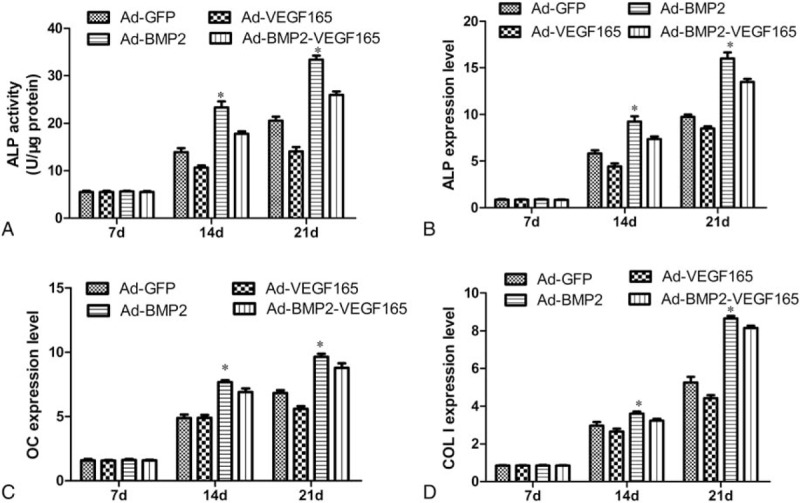
Detection of ALP activity and analysis of mRNA of osteogenic genes in vitro for each group seeded in osteogenic differentiation medium at 7, 14, and 21 days. (A) Quantitative analysis of ALP activity. The BMP2 group showed markedly ALP activity at 14 and 21 days. ∗*P* < .05 compared with BMP2+VEGF165 group, VEGF165 group, and GFP group. Each assay was performed in triplicate. (B–D) The osteogenesis genes expression of ALP, OC Col I in BMP2 group showed markedly increased osteogenesis genes level. ∗*P* < .05 compared with BMP2 +VEGF165 group, VEGF165 group, and GFP group. ALP = alkaline phosphatase, BMP2 = bone morphogenetic protein 2, Col I = collagen type I, OC = osteocalcin, VEGF165 = vascular endothelial growth factors 165.

As shown in Figure 5, ALP activity peaked at 21 days in all of the groups. ALP activity in the BMP2 group and BMP2+VEGF group increased on average by 2.5-fold and 1.7-fold compared to the VEGF165 group at the 21 days, respectively. Meanwhile, ALP activity in controls was higher than that in VEGF165 group at the 14 and 21 days (*P* < .05). There was no significant difference between control group and VEGF165 group at 7 days.

### 3.4 Osteoblastic gene expression in vitro

Osteoblasts were assessed for ALP, OC, and Col I at 7, 14 and 21 days post-transfection. Gene expression in each group increased time dependently and reached a peak at 21 days. Gene expression in the BMP2 group and BMP2+VEGF group showed significantly higher transcriptional levels at 14 and 21 days than the others. VEGF165 gene expression did not promoted osteogenesis in the VEGF165 group and BMP2+VEGF groups. By contrast, osteoblastic genes in the BMP2 group expressed higher expression levels, and have a significant difference compared to VEGF group and control groups. At 14 and 21 days, the ALP activity of BMP2+VEGF165 group was highly suppressed compared with that of BMP2 group (*P* < .05). However, there were no differences between the groups at 7 days (Fig. 5)

## 4 Discussion

Bone regeneration is a complex progress involving osteogenesis and angiogenesis, Synergy of BMP2 and VEGF165 during osteogenesis and angiogenesis stimulates bone regeneration and bone remolding.^[14]^ BMP2, a potent osteogenic protein promotes proliferation, migration, and osteoblast differentiation of BMSCs.^[15]^ Recombinant human BMP2 enhances bone repair in animal models^[16,17]^ and is approved by the US Food and Drug Administration for use in absorbable collagen sponges for interbody fusion of the lumbar spine^[18]^.

Peterson et al^[19]^ reported that BMP2 exogene transduction in BMSCs can heal critically sized femoral defects in mouse models. However, genetically modified BMSCs expression BMP2 was less effective in repairing critical bone defects because of an insufficient blood vessel network in the scaffold.^[20]^ Nowadays, researchers focus on vascularization in bone regeneration tissue vessel-induced factors like VEGF165. VEGF165 plays an important role in bone regeneration via the endochondral ossification pathway. Interdicting VEGF leads to a decrease in bone formation by suppressing blood vessel invasion and cartilage resorption.^[21]^ VEGF165 has been shown to participate in bone formation and reabsorption through osteoblasts and osteoclasts.

Because of inadequate blood vessel formation in tissue, about 25% of nonunion fractures need a secondary bone graft procedure after single BMP2 repair.^[22]^ Meanwhile, the beneficial effect of bone formation depends critically on the ratio of VEGF to BMP4 (1:5) and an inappropriate ratio of these factors leads to detrimental effects on bone regeneration.^[3]^

The effect of VEGF165 on MSCs differentiation into osteoblasts has recently been widely discussed. Some reports demonstrate that overexpressed VEGF inhibited BMP2-induced MSCs differentiation and osteogenesis in vitro.^[23,24]^ To offer an efficient strategy to repair bone defects, it is necessary to research and understand the molecular interaction of VEGF165 and BMP2 on BMSCs differentiating to osteoblasts in vitro.

In the present study, BMSCs were selected as subjects because they are dominant seed cells for bone engineering and possess the ability to differentiate into osteoblasts, chondrocyte, neurocyte and among others when supplied to or co-cultured with different types of agents and growth factors.^[25]^

We show that VEGF165 can inhibit the osteoblastic differentiation of BMSCs in vitro. However, we could not find the promoting or inhibitory effect between BMP2 and VEGF165 using ELISA and Western Blot. The expression from T2A sequence subcloned in adenovirus vector to link the 2 interest genes and no one group co-containing Ad-BMP2 and Ad-VEGF165 observed were the main reasons. Although a significant difference between the groups was detectable by OC staining at 21 days in vitro, inhibition is evident in the VEGF and BMP2+VEGF165 groups at 14 and 21 days, respectively, through the assay of ALP activity and RT-PCR.

A similar conclusion was revealed by some other studies. Schonmeyr et al^[11]^ showed that Ad-VEGF and human recombinant VEGF inhibit BMP2 mRNA expression and protein production at the transcriptional and translation levels. The effect is concentration-dependently related to VEGF, and inhibition occurs through autocrine as well as paracrine mechanisms. Lin et al ^[23]^ showed that BMP2 exerts a stronger effect on bone formation than combined BMP2 and VEGF165 as detected the alizarin red and ALP staining. Song et al^[9]^ demonstrated that overexpression of Id I gene retards terminal osteoblast differentiation induced by both BMP2 and VEGF165. OC and ALP activity gradually decreased in vitro, and smaller bone formation was detected in Ad-BMP2-BMSC+Ad-VEGF-EPC group in vivo. Geiger et al^[26]^ revealed that the carrier loaded with BMSCs showed the highest degree of osteogenesis and the highest vascularization and faster resorption of the carrier was found in VEGF-transfected cells group. Similar conclusion were reached by Peng's et al^[3]^ study, who reported that VEGF alone did not sufficiently promote bone regeneration, it acted synergistically with BMP4 to enhance cell survival, and increased MSCs recruitment and bone formation. The effect of VEGF on the regulation of bone homeostasis is related to other factors, including blood vessel invasion.^[27]^

## 5 Conclusion

We used the adenovirus vector as the exogene transduction vector to efficiently and stably co-express BMP2 and VEGF165. Our data show that overexpression of VEGF165 inhibits BMSCs and BMP2-induced BMSCs differentiation into osteoblasts in vitro. Meanwhile, application of BMP2 alone demonstrated a strong effect on osteogenesis in vitro. Our experiments establish a foundation to explore the biological effects of single and combined BMP2 and VEGF165 in vitro. However, various growth factors besides BMP2 are coordinated by and involved in osteogenesis. The mechanism and inhibition effect of VEGF165 on bone regeneration in vivo remain to be elucidated.

## References

[1] Nguyen BB Moriarty RA Kamalitdinov T Collagen hydrogel scaffold promotes mesenchymal stem cell and endothelial cell coculture for bone tissue engineering,. J Biomed Mater Res A 2017;105:1123–31. 28093887 10.1002/jbm.a.36008 PMC5328802

[2] Brett E Flacco J Blackshear C Biomimetics of bone implants: the regenerative road. Biores Open Access 2017;6:1–6. 28163982 10.1089/biores.2016.0044 PMC5248549

[3] Peng H Wright V Usas A Synergistic enhancement of bone formation and healing by stem cell-expressed VEGF and bone morphogenetic protein-4. J Clin Invest 2002;110:751–9. 12235106 10.1172/JCI15153 PMC151123

[4] Kiyozuka Y Miyazaki H Yoshizawa K An autopsy case of malignant mesothelioma with osseous and cartilaginous differentiation: bone morphogenetic protein-2 in mesothelial cells and its tumor. Dig Dis Sci 1999;44:1626–31. 10492144 10.1023/a:1026627413715

[5] Egri S Eczacioglu N Sequential VEGF and BMP-2 releasing PLA-PEG-PLA scaffolds for bone tissue engineering: I. Design and in vitro tests. Artif Cells Nanomed Biotechnol 2017;45:321–9. 26912262 10.3109/21691401.2016.1147454

[6] Ferrara N Vascular endothelial growth factor and the regulation of angiogenesis. Recent Prog Horm Res 2000;55:15–35. discussion 35-16. 11036931

[7] Wang Y Wan C Deng L The hypoxia-inducible factor alpha pathway couples angiogenesis to osteogenesis during skeletal development. J Clin Invest 2007;117:1616–26. 17549257 10.1172/JCI31581 PMC1878533

[8] Liu B Li X Liang G VEGF expression in mesenchymal stem cells promotes bone formation of tissue-engineered bones. Mol Med Rep 2011;4:1121–6. 21850376 10.3892/mmr.2011.559

[9] Song X Liu S Qu X BMP2 and VEGF promote angiogenesis but retard terminal differentiation of osteoblasts in bone regeneration by up-regulating Id1. Acta Biochim Biophys Sin (Shanghai) 2011;43:796–804. 21880603 10.1093/abbs/gmr074

[10] Sharmin F McDermott C Lieberman J Dual growth factor delivery from biofunctionalized allografts: sequential VEGF and BMP-2 release to stimulate allograft remodeling. J Orthop Res 2016;35:1086–95. 10.1002/jor.23287 27155087

[11] Schonmeyr BH Soares M Avraham T Vascular endothelial growth factor inhibits bone morphogenetic protein 2 expression in rat mesenchymal stem cells. Tissue Eng Part A 2010;16:653–62. 19754224 10.1089/ten.tea.2009.0426 PMC2947933

[12] Li G Corsi-Payne K Zheng B The dose of growth factors influences the synergistic effect of vascular endothelial growth factor on bone morphogenetic protein 4-induced ectopic bone formation. Tissue Eng Part A 2009;15:2123–33. 19215221 10.1089/ten.tea.2008.0214 PMC2811054

[13] Eca LP Ramalho RB Oliveira IS Comparative study of technique to obtain stem cells from bone marrow collection between the iliac crest and the femoral epiphysis in rabbits. Acta Cir Bras 2009;24:400–4. 19851694 10.1590/s0102-86502009000500011

[14] Zhang C Wang KZ Qiang H Angiopoiesis and bone regeneration via co-expression of the hVEGF and hBMP genes from an adeno-associated viral vector in vitro and in vivo. Acta Pharmacol Sin 2010;31:821–30. 20581855 10.1038/aps.2010.67 PMC4007728

[15] Chen D Zhao M Mundy GR Bone morphogenetic proteins. Growth Factors (Chur, Switzerland) 2004;22:233–41. 10.1080/08977190412331279890 15621726

[16] Kolambkar YM Dupont KM Boerckel JD An alginate-based hybrid system for growth factor delivery in the functional repair of large bone defects. Biomaterials 2011;32:65–74. 20864165 10.1016/j.biomaterials.2010.08.074 PMC3013370

[17] Oest ME Dupont KM Kong HJ Quantitative assessment of scaffold and growth factor-mediated repair of critically sized bone defects. J Orthop Res 2007;25:941–50. 17415756 10.1002/jor.20372

[18] Einhorn TA Clinical applications of recombinant human BMPs: early experience and future development. J Bone Joint Surg Am 2003;85-A (suppl 3):82–8. 10.2106/00004623-200300003-00014 12925614

[19] Peterson B Zhang J Iglesias R Healing of critically sized femoral defects, using genetically modified mesenchymal stem cells from human adipose tissue. Tissue Eng 2005;11:120–9. 15738667 10.1089/ten.2005.11.120

[20] Xiao C Zhou H Liu G Bone marrow stromal cells with a combined expression of BMP-2 and VEGF-165 enhanced bone regeneration. Biomed Mater (Bristol, England) 2011;6:015013. 10.1088/1748-6041/6/1/015013 21252414

[21] Duan X Bradbury SR Olsen BR VEGF stimulates intramembranous bone formation during craniofacial skeletal development. Matrix Biol 2016;52–54:127–40. 10.1016/j.matbio.2016.02.005 PMC4875795 26899202

[22] Kujala S Raatikainen T Ryhanen J Composite implant of native bovine bone morphogenetic protein (BMP) and biocoral in the treatment of scaphoid nonunions—a preliminary study. Scand J Surg 2002;91:186–90. 12164521 10.1177/145749690209100210

[23] Lin Z Wang JS Lin L Effects of BMP2 and VEGF165 on the osteogenic differentiation of rat bone marrow-derived mesenchymal stem cells. Exp Ther Med 2014;7:625–9. 24520257 10.3892/etm.2013.1464 PMC3919869

[24] Kuroda S Sumner DR Virdi AS Effects of TGF-beta1 and VEGF-A transgenes on the osteogenic potential of bone marrow stromal cells in vitro and in vivo. J Tissue Eng 2012;3: 2041731412459745. 10.1177/2041731412459745 PMC3434762 22962632

[25] Reddi AH Bone and cartilage differentiation. Curr Opin Genet Dev 1994;4:737–44. 7849513 10.1016/0959-437x(94)90141-o

[26] Geiger F Lorenz H Xu W VEGF producing bone marrow stromal cells (BMSC) enhance vascularization and resorption of a natural coral bone substitute. Bone 2007;41:516–22. 17693148 10.1016/j.bone.2007.06.018

[27] Wang DS Yamazaki K Nohtomi K Increase of vascular endothelial growth factor mRNA expression by 1,25-dihydroxyvitamin D3 in human osteoblast-like cells. J Bone Miner Res 1996;11:472–9. 8992878 10.1002/jbmr.5650110408

